# The Opportunities of the Wealthy

**Published:** 1903-12-26

**Authors:** 


A Journal of

The Medical Sciences and Hospital Administration.

Vol. XXXV.—No. 900. DECEMBER 26, 1903.

### The Hospital.

### THE OPPORTUNITIES OF THE WEALTHY.

The Christmas season is traditionally that at which the purse-strings of the wealthy are expected to be somewhat more relaxed than usual, and at which those who are possessed of a superabundance of this world's goods may be supposed to be making some examination of the respective validity of the many claims which are practically certain to be urged upon them. In these there is at least a sufficiency of variety. The Prince of Wales has lately pointed out that the extraordinary demands to be expected from London hospitals during the coming year will certainly exceed a million sterling; and yet it is by no means impossible that there may be people who, in Scottish phrase, will be so "left to themselves" as to devote a portion of their superfluities to the payment of the costs and damages incurred by a libeller whose chief object in life seems to be to prevent the work of hospitals from being carried on. Among objects of a more legitimate kind may be found associations and societies for the amelioration of nearly every evil to which flesh is heir; and as to some of which, indeed, the wisdom of the founders is as little assured as the efficacy of the remedial measures which they propose to bring into operation. A rich man who desires to do some good to humanity with his money will often find himself embarked in a business which requires all his sagacity to carry it to a successful issue.

Among the opportunities for benevolent expenditure now being urged upon the public it is perhaps possible to draw a line separating two great classes of undertakings, those which seek for the supply of their current and constantly recurring demands, and those which seek for endowments by which their stability and efficiency may be permanently secured. It may be perfectly wise and right for a millionaire to limit his donation to a charity which may be described as a "going concern" to some reasonable contribution towards the expenses of the year, leaving it to others, from time to time, to provide for similar necessities as they arise. But there are conditions in which donations of such a kind are but of small utility, and in which the necessities of the case can only be met by gifts of such amount as either to provide for extraordinary requirements in the present or to afford security for the future. Two of such claims have at this time come conspicuously before the public, in the shape of the demand for the reconstruction of St. Bartholomew's Hospital, and the demand for the endowment of the Medical Faculty of the University of London. Of the former it is almost superfluous to speak, but we may be excused for directing attention to the latter.

There is only too much reason to believe, as we have more than once had occasion to suggest, that the scientific and preliminary education of medical students has for some years been upon a downward grade, and that the imperfection of the means provided for conducting it, and the inadequacy of the examinations by which it has been tested, have continually acted and reacted upon each other. Not only has this been true of the transparent sham of scientific teaching conducted in common schools and accepted by the English Royal Colleges and by the Society of Apothecaries, but also, and probably in a still greater degree, of the teaching of the medical curriculum. The position of a teacher of physiology at some of the London schools can only be described as heart-breaking. The small class afforded him by the scale of entries of recent years has been insufficient to meet the bare cost of the necessary apparatus and experiments, and insufficient, *a fortiori*, to afford him any remuneration for his labour. Many of the schools, as is well known, have altogether failed to pay their expenses, and have only been enabled te keep open their doors by a subsidy from the funds of the hospital to which they are attached. Upon such, at least as regards their preliminary departments, the stamp of perfunctoriness must almost of necessity be impressed, even if the clinical instruction be such as to fulfil all reasonable requirements, and it is accordingly found that the provincial schools, by a greater concentration of their efforts, and by combining the clinical students from different hospitals for the purposes of preliminary teaching, are very rapidly coming to the front, and have already deprived the metropolis of the pre-eminent position as a school of medicine which it once enjoyed, and which, by reason of its superabundance of medical and surgical skill and knowledge, and of its unparalleled wealth of clinical material, it certainly should be assisted to retain. If we were beginning with a clean sheet, and with the advantage of modern facilities for locomotion, it might be possible for the 11 medical schools of London to combine, and to establish a single school for preliminary subjects, adequately equipped with teachers and with apparatus, and affording adequate remuneration to the former. Such an arrangement, however desirable, is now only possible by the aid of an initiative from without; and it is for the accomplishment of this purpose that the University of London seeks such an endowment of its medical faculty as may enable it to give instruction in anatomy, physiology, and related subjects, on a scale and in a manner commensurate with their importance, and may thus not only relieve the hospital schools from a burden which has been proved to be beyond their powers, but may also provide them with clinical students sufficiently well trained and taught to be able to appreciate and to use the opportunities which will be presented to them in the wards. We are glad to see that Lord Rosebery, as Chancellor of the University, has put his hand to the plough in relation to this matter, and we earnestly hope that his furrow may no longer be a lonely one. The question is one of prodigious importance alike to the progress of medicine and to the welfare of the sick; and we question whether a millionaire could find any equally advantageous opportunity for raising to himself a *monumentum ære perennius.*

